# Effect of Stay-Green Wheat, a Novel Variety of Wheat in China, on Glucose and Lipid Metabolism in High-Fat Diet Induced Type 2 Diabetic Rats

**DOI:** 10.3390/nu7075143

**Published:** 2015-06-26

**Authors:** Jinshan Ji, Chao Zhang, Xiaoqin Luo, Li Wang, Ruijuan Zhang, Zhenlin Wang, Daidi Fan, Haixia Yang, Jianjun Deng

**Affiliations:** 1 Nutrition and Food Safety Engineering Research Center of Shaanxi Province, Department of Public Health, School of Medicine, Xi’an Jiaotong University, Xi’an 710061, China; E-Mails: yadxjjs@163.com (J.J.); yyyyyy_214@163.com (C.Z.); miniqiao@126.com (X.L.); zhangrj@mail.xjtu.edu.cn (R.Z.); zlwd@mail.xjtu.edu.cn (Z.W.); 2 Cardiovascular Research Center, Xi’an Jiaotong University, Xi’an 710061, China; 3 Medical College, Yan’an University, Yan’an 716000, China; E-Mail: yadxwl0209@163.com; 4 Shaanxi Key laboratory of Degradable Biomedical Materials, Department of Food Science and Engineering, College of Chemical Engineering, Northwest University, Xi’an 710069, China; E-Mail: fandaidi@nwu.edu.cn

**Keywords:** stay-green wheat, type 2 diabetic mellitus, high-fat diet, antioxidant activities

## Abstract

The use of natural hypoglycemic compounds is important in preventing and managing Type 2 diabetes mellitus (T2DM). Forty male Sprague-Dawley rats weighing 150–180 g were divided into four groups to investigate the effects of the compounds in stay-green wheat (SGW), a novel variety of wheat in China, on T2DM rats. The control group (NDC) was fed with a standard diet, while T2DM was induced in the rats belonging to the other three groups by a high-fat diet followed by a streptozotocin (STZ) injection. The T2DM rats were further divided into a T2DM control group (DC), which was fed with the normal diet containing 50% common wheat flour, a high dose SGW group (HGW) fed with a diet containing 50% SGW flour, and a low dose SGW group (LGW) fed with a diet containing 25% SGW flour and 25% common wheat flour. Our results showed that SGW contained cereal antioxidants, particularly high in flavonoids and anthocyanins (46.14 ± 1.80 mg GAE/100 g DW and 1.73 ± 0.14 mg CGE/100 g DW, respectively). Furthermore, SGW exhibited a strong antioxidant activity *in vitro* (30.33 ± 2.66 μg TE/g DW, *p* < 0.01). Administration of the SGW at a high and low dose showed significant down-regulatory effects on fasting blood glucose (decreasing by 11.3% and 7.0%, respectively), insulin levels (decreasing by 12.3% and 9.7%, respectively), and lipid status (decreasing by 9.1% and 7.5%, respectively) in T2DM rats (*p* < 0.01). In addition, the T2DM groups treated with SGW at a high and low dose showed a significant increase in the blood superoxide dismutase (1.17 fold and 1.15 fold, respectively) and glutathione peroxidase activities (1.37 fold and 1.30 fold, respectively) compared with the DC group (*p* < 0.01). The normalized impaired antioxidant status of the pancreatic islet and of the liver compared with the DC group was also significantly increased. Our results indicated that SGW components exerting a glycemic control and a serum lipid regulation effect may be due to their free radical scavenging capacities to reduce the risk of T2DM in experimental diabetic rats.

## 1. Introduction

Diabetes mellitus is a chronic metabolic disorder that represents a major and growing public health problem all over the world. Diabetes mellitus incidence increased from approximately 100–135 million affected adults worldwide in 1995 to an estimated 285 million in 2010 [1]. Type 2 diabetes mellitus (T2DM), that represents the predominant type of diabetes mellitus, is characterized by a chronic hyperglycemia and a marked lipid metabolic disorder [2]. It may result from genetic or several environmental factors, such as high-fat diet and less exercise. Several factors are associated with the pathology of diabetes leading to the dysregulation of both glucose and lipid metabolism. Among these, oxidative stress due to free radicals production, such as increased O^2−^ from mitochondria, affects and impairs cell membranes and results in insulin resistance, β-cell dysfunction, impaired glucose tolerance and finally T2DM [3]. Antioxidants possess the ability to scavenge free radicals and reduce the deleterious consequences that affect the lipids, regulating the oxidative stress-sensitive signaling pathways. Thus, their use in T2DM patients is widely considered as an efficient method to alleviate diabetes and its complications [3].

A diet containing a high amount of natural antioxidants may represent a safe and effective method for the control and prevention of T2DM [4]. Many *in vitro* and *in vivo* studies demonstrated that frequent consumption of whole grain foods improved metabolic homeostasis and reduced the risk of T2DM and its complications [4,5]. Some whole grain foods, such as wheat [6,7], barley [8,9] and oat [10], contain relative high amounts of phytochemicals such as phenolic compounds, phytosterols and folate, widely known as powerful antioxidants able to control and prevent T2DM by reducing oxidative stress. In addition, phenolic compounds represent the most diverse and complex class of phytochemicals considered as the major contributors to the total antioxidant activity of cereal grains [6,11]. Stay-green wheat (*Triticum aestivum* L.) (SGW), which is called “Lvfeng 1”, is a mutant type of the common wheat, with delayed leaf senescence because of an extended duration of the active photosynthesis during the grain-filling period [12,13,14]. SGW maintains a relatively high antioxidant enzyme activity and resistance to photo-oxidative stress [12,14], but little is known about the type and concentration of the active phytochemical components of SGW grains and their antioxidant properties. Additionally, the relevant medicinal and therapeutic properties of SGW, as well as its potential role on health improvement, have not yet been reported. Hence, the aim of the present study was to analyze the active components content of SGW and their antioxidant activities *in vivo* and *in vitro* to evaluate their alleviative effects on glucose and lipid metabolic disorders in high-fat diet and streptozotocin induced T2DM rats.

## 2. Experimental Section

### 2.1. Materials

SGW was kindly provided by Shaanxi Houji Featured Agriculture R&D Centre. All chemicals used were purchased from Sigma-Aldrich (St. Louis, MO, USA) unless otherwise indicated.

### 2.2. Determination of the Phytochemical Content and Total Antioxidant Activity in Vitro

The oxygen radical absorbance capacity (ORAC) assay was performed according to the method previously reported [15] and the results were expressed as μmol Trolox equivalent (TE) per g dry weight. Total phenolic contents in the wheat grain were measured using Folin-Ciocalteu reagent as reported [16] and expressed as milligrams of gallic acid equivalent (GAE) per 100 g dry weight. Total flavonoid contents were determined by a colorimetric method as previously described [15] and expressed as milligrams of catechin equivalents (CE) per 100 g dry weight. Total anthocyanins were determined as described [17] and expressed as milligrams of cyaniding-3-glucoside equivalent (CGE) per 100 g dry weight. Phenolic compositions were identified on the basis of their characteristic UV-Vis spectra and retention times by the HPLC method [18]. Results were confirmed by the peaks using synthetic syringic acid, vanillic acid, caffeic acid and ferulic acid, and quantified with an external calibration curve with the corresponding standards.

### 2.3. Animal Treatment

The animal experiment was performed within the jurisdictional framework of the Animal Management Rules of the Ministry of Health of China and the guidelines for the Care and Use of Laboratory Animals of Xi’an Jiaotong University (approval number XJTULAC2012-012; 10 February 2012). Forty male Sprague-Dawley (SD) rats weighing 150–180 g were provided by the Experimental Animal Center of Xi’an Jiaotong University and housed in individual cages exposed to a 12 h light/dark cycle. They were allowed free access to water and a hand-made standard pelleted diet (common flour 50%, bran 13%, whey powder 1%, fish oil 7%, bean pulp 26%, salt 0.5%, calcium bicarbonate 1.4%, minerals 0.5% and vitamins 0.6%). After five days of adaptation, glucose and lipid levels in the blood taken from the tail vein were measured. The non-diabetic control group (NDC) consisting of 9–10 rats, was fed the standard pelleted diet. To induce T2DM, rats were fed a high-fat diet (10% lard, 20% sucrose, 10% yolk powder, 0.2% sodium deoxycholate, and 1% cholesterol combined with 59% standard diet) for seven weeks. Subsequently, rats were fasted overnight and the following morning they received a single intraperitoneal injection of streptozotocin (STZ) diluted in citrate buffer (pH 4.0) at a dose of 30 mg/kg bodyweight. After 72 h, the blood glucose levels were determined by collecting blood from the rats’ tail vein. The rats with glycemia >16.8 mmol/L were considered as T2DM. The T2DM rats were randomly divided into three groups, each consisting of 10 animals. One group of rats was the T2DM control (DC) and was fed the standard diet and the other two experimental groups were fed, respectively, the diet containing 50% SGW flour (HGW, 116.67 g/kg·day) and the diet containing 25% SGW flour plus 25% common wheat flour (LGW, 58.33 g/kg·day). Food and water intake were determined once a day by estimating the amount consumed. After eight weeks of administration, blood samples were collected from the inferior vena cava of the anesthetized rats. Then, cardiac puncture was carried out, and the pancreas and liver were harvested and stored in 10% formaldehyde solution for two weeks and then transferred and kept in 80% ethyl alcohol until histopathological analyses.

### 2.4. Oral Glucose Tolerance Test (OGTT)

A blood sample was taken at 0 min from the tip of the tail. Next, d-glucose (2 g d-glucose/kg body weight dissolved in 0.9% saline) was orally administered by gavage. Rats were fasted for 12 h before the OGTT.

### 2.5. Insulin and Homeostatic Model Assessment-Insulin Resistance (HOMA-IR) Determination

Insulin levels were quantified using rat insulin enzyme linked immunosorbent assay (ELISA) kits (Crystal Chem, Downers Grove, IL, USA). HOMA-IR was calculated using the formula HOMA-IR = (glucose × insulin)/22.5, where the concentration of glucose is expressed in mmol/L and that of insulin in mIU/L [5].

### 2.6. Blood Biochemical Analysis

According to the standard methods, serum glucose and lipid components such as total cholesterol (TC), triglyceride (TG), high-density lipoprotein cholesterol (HDL-C), low-density lipoprotein cholesterol (LDL-C), non-esterified fatty acids (NEFA), superoxide dismutase (SOD) and glutathione peroxidase (GSH-px) were measured by colorimetric enzyme kits according to the manufacturer’s protocols (Sigma-Aldrich). The SOD and GSH-px activities were defined as the amount of enzymatic reaction of 1 mL serum per minute.

### 2.7. Histopathological Examination

The pancreas and liver tissues were fixed in 10% buffered formalin and embedded in paraffin. The paraffin-fixed tissue specimens were sliced into 4 μm-thick sections. The sections were mounted on glass slides and stained with Hematoxylin and Eosin (HE staining) and examined with a light microscopy.

### 2.8. Statistical Analysis

Quantitative data are expressed as mean ± SEM. Student’s *t*-test was used to compare the changes in phytochemical contents and total antioxidant activity in both groups. One-way analysis of variance (ANOVA) followed by Tukey’s *post hoc* test was used to compare changes in water intake, blood insulin and lipids status, and oxidative stress parameter levels of these four experimental groups. Two-way repeated measures ANOVA followed by the Bonferroni multiple comparisons test was used to identify the factors “treatment” and “time” and to analyze their interactions (treatment × time) influencing the body weight, food intake, and blood glucose levels (SPSS 19.0 for Windows, IBM, Chicago, IL, USA). *p* < 0.05 was considered statistically significant. Non-quantitative results were derived from at least three independent experiments.

## 3. Results

### 3.1. SGW Phytochemical Content and Total Antioxidant Activity in Vitro

SGW total antioxidant activity *in vitro* was measured by the ORAC method (Table 1). SGW exhibited a stronger and significant antioxidant activity (30.33 ± 2.66 μg TE/g DW) compared with the common wheat (24.12 ± 1.03 μg TE/g DW) (*p* < 0.05). Moreover, the SGW analysis showed that the total flavonoid content (46.14 ± 1.80 mg GAE/100 g DW) was more than twice the content in the common wheat (20.25 ± 0.78 mg GAE/100 g DW), while the total phenolic content (88.82 ± 5.91 GAE/100 g DW) was similar with the content in the common wheat (85.84 ± 3.02 mg GAE/100 g DW). Total anthocyanin content in the SGW was 1.73 ± 0.14 mg CGE/100 g DW, while anthocyanins were not detected in the common wheat. The analysis of the phenolic composition showed that SGW was rich in vanillic acid (74.54 ± 6.41 μg/g) compared with the common wheat (22.23 ± 0.93 μg/g).

**Table 1:** Total phenolic, total flavonoid, total anthocyanin content and phenolic composition of SGW.

	SGW	Common Wheat
Total antioxidant activity (ORAC) (μg TE/g DW)	30.33 ± 2.66 ^†^	24.12 ± 1.03
Total phenolic (mg GAE/100 g DW)	88.82 ± 5.91	85.84 ± 3.02
Total flavonoid (mg CE/100 g DW)	46.14 ± 1.80 ^†^	20.25 ± 0.78
Total anthocyanin (mg CGE/100 g DW)	1.73 ± 0.14	nd
**Phenolic Composition**		
Syringic acid (μg/g)	7.16 ± 0.63 ^†^	36.39 ± 1.91
Vanillic acid (μg/g)	74.54 ± 6.41 ^†^	22.23 ± 0.93
Caffeic acid (μg/g)	3.08 ± 0.57 ^†^	7.87 ± 0.61
Ferulic acid (μg/g)	3.70 ± 0.89	2.51 ± 0.80

nd: not detected; results were expressed as means ± SEM; ^†^ *p* < 0.05 *vs.* common wheat.

### 3.2. Rats Body Weight, Food and Water Intakes

**Figure 1:**
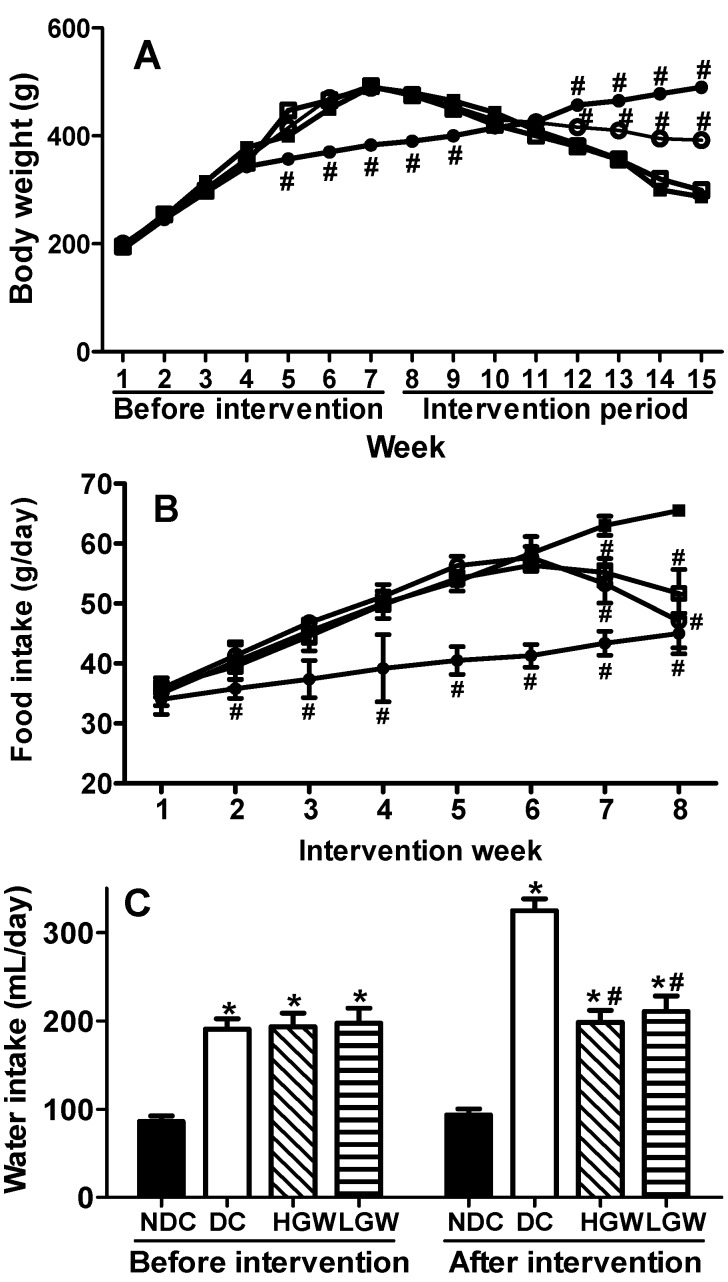
Metabolic analyses in rats. (**A**) Body weight measured during the whole feeding period. (**B**) Food intake measured during the SGW intervention. ●, NDC group; ■, DC group; ○, HGW group; □, LGW group. According to two-way repeated measures ANOVA, there was a significant interaction between the effects of treatment and time on body weight (*p* < 0.01) and food intake (*p* < 0.01), respectively. (**C**) Water intake was measured before and after the intervention. Results were expressed as means ± SEM (*n* = 9–10). * *p* < 0.01 *vs.* NDC group, # *p* < 0.01 *vs.* DC group.

The body parameters such as body weight, food and water intakes, are shown in Figure 1. Figure 1A shows the changes in body weight during the entire feeding period. Two-way repeated measure ANOVA results indicated a significant treatment × time interaction positively associated with body weight (*p* < 0.01). The rats belonging to the three groups fed with a high-fat diet significantly increased their body weights compared with the rats in the NDC group (*p* < 0.01) before the SGW intervention. However, the rats in the T2DM groups showed a remarkable weight decrease soon after the start of the SGW intervention, while the weight of the rats in the NDC group continued to increase (*p* < 0.01). Additionally, the weight of the rats in the HGW group was decreased compared with the weight of the rats in the DC group (*p* < 0.01), while the weight difference between the rats in the LGW and HGW group was not significant. As shown in Figure 1B, two-way ANOVA also demonstrated a significant interaction between treatment and time on food intake (*p* < 0.01). Both the food and water intake results showed that the rats in the T2DM groups ate and drank more than the rats in the NDC group (*p* < 0.01) (Figure 1B,C). However, the food and water intake of the rats in the intervention groups significantly decreased to the normal level in the last two weeks during the intervention period when compared with the intake of the rats in the DC group (*p* < 0.01).

### 3.3. Blood Glucose and Insulin Level Changes

**Figure 2:**
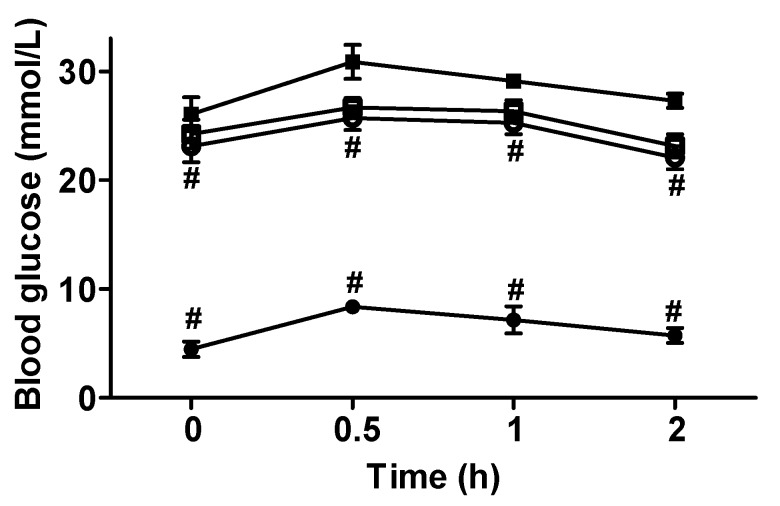
Glucose tolerance test of the rats in different groups after stay-green wheat (SGW) intervention. ●, NDC group; ■, DC group; ○, HGW group; □, LGW group. According to two-way repeated measures ANOVA, there was a significant interaction between the effects of treatment and time on blood glucose (*p* < 0.01). Results were expressed as means ± SEM (*n* = 9–10). # *p* < 0.01 *vs.* DC group.

According to the two-way repeated measure ANOVA results, a significant treatment × time interaction positively associated with blood glucose was observed (*p* < 0.01). Figure 2 shows that the fasting glucose level in the T2DM rats was higher than the level in the rats of the NDC group. The areas under the OGTT curves belonging to the diabetes rat groups (87.32 ± 2.01 for DC group, 73.55 ± 1.58 for HGW group and 76.71 ± 1.32 for LGW group) were significantly higher than the areas belonging to the NDC group (20.64 ± 1.03) (*p* < 0.01). The fasting glucose level in the HGW and LGW groups significantly decreased when compared with the level in the DC group (*p* < 0.01), but no difference was found between the two treatment groups. The fasting insulin level and the homeostasis model assessment-insulin resistance index (HOMA-IR) showed the same trend of the OGTT curves (Table 2).

**Table 2:** Blood glucose level in the rats of different groups after stay-green wheat (SGW) intervention.

	Serum Insulin (mIU/L)	HOMA-IR
NDC	11.42 ± 0.23	2.78 ± 0.21
DC	24.56 ± 0.17 *	23.89 ± 1.48 *
HGW	21.54 ± 0.36 ^#^	16.83 ± 0.46 ^#^
LGW	22.17 ± 0.22 ^#^	18.80 ± 0.63 ^#^

NDC, control group; DC, T2DM control group; HGW, high dose stay-green wheat (SGW) group; LGW, low dose SGW group; results were expressed as means ± SEM (*n* = 9–10); * *p* < 0.01 *vs.* NDC group; ^#^ *p* < 0.01 *vs.* DC group.

### 3.4. Blood Lipid Changes

The serum lipid parameters in the fasting condition, such as TC, TG, LDL-C, and NEFA in the T2DM rats were significantly increased while HDL-C was significantly decreased compared with the NDC group (*p* < 0.01) (Table 3). Both HGW and LGW groups improved their lipid status except HDL-C after the SGW feeding, showing a statistically significant difference in the level of the serum lipid parameters compared with the DC group (*p* < 0.01). However, no significant difference was observed between HGW and LGW groups.

**Table 3:** Blood lipid profile in the rats of different groups after stay-green wheat (SGW) intervention.

	TC (mg/dL)	TG (mg/dL)	HDL-C (mg/dL)	LDL-C (mg/dL)	NEFA (mequiv/L)
NDC	65.27 ± 0.47	84.07 ± 3.54	26.47 ± 0.50	38.62 ± 0.42	0.37 ± 0.04
DC	92.14 ± 0.53 *	286.73 ± 4.47 *	19.18 ± 1.15 *	63.02 ± 0.49 *	1.03 ± 0.07 *
HGW	83.74 ± 0.63 ^#^	252.21 ± 7.77 ^#^	21.12 ± 0.39	57.18 ± 0.41 ^#^	0.70 ± 0.06 ^#^
LGW	85.23 ± 0.32 ^#^	267.25 ± 7.34 ^#^	20.34 ± 0.48	60.73 ± 0.50 ^$^	0.82 ± 0.07 ^$^

TC, total cholesterol; TG, triglyceride; HDL-C, high-density lipoprotein cholesterol; LDL-C; low-density lipoprotein cholesterol; NEFA, non-esterified fatty acids; results were expressed as means ± SEM (*n* = 9–10); * *p* < 0.01 *vs.* NDC group; ^$^ *p* < 0.05 *vs.* DC group; ^#^ *p* < 0.01 *vs.* DC group.

### 3.5. Antioxidant Effects

Table 4 showed the effects of SGW against oxidative stress in diabetes rats. Both SOD and GSH-Px activities in the DC groups were decreased compared with the NDC group (*p* < 0.01). After the administration of high and low doses of SGW, the SOD activities were increased from 148.78 in the DC group to 174.62 (*p* < 0.01) and 171.00 U/mL (*p* < 0.05) in the HGW and LGW groups, respectively. Moreover, increased GSH-Px activities were also observed in the HGW and LGW groups compared with the DC group (1.37 and 1.30 fold more than the DC group, respectively, *p* < 0.01). However, no changes were found between the HGW and LGW groups.

**Table 4:** Oxidative stress parameter levels in the rats of different groups after stay-green wheat (SGW) intervention.

	SOD (U/mL)	GSH-Px (U/mL)
NDC	207.50 ± 12.19	537.00 ± 8.35
DC	148.78 ± 4.53 *	298.67 ± 11.05 *
HGW	174.62 ± 7.47 ^$^	408.87 ± 9.94 ^#^
LGW	171.00 ± 6.24 ^#^	389.56 ± 8.43 ^#^

SOD, superoxide dismutase; GSH-Px, glutathione peroxidase; results were expressed as means ± SEM (*n* = 9–10); * *p* < 0.01 *vs.* NDC group; ^$^ *p* < 0.05 *vs.* DC group; ^#^ *p* < 0.01 *vs.* DC group.

### 3.6. Histological Changes

**Figure 3:**
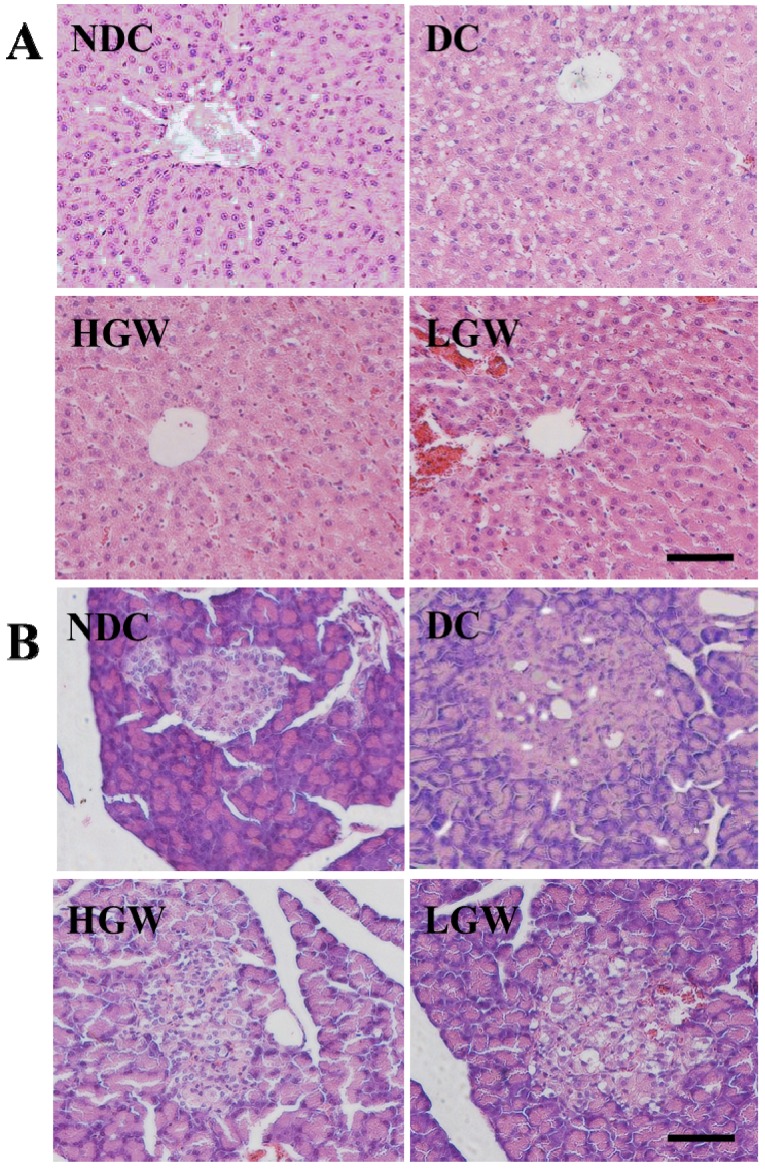
Effect of stay-green wheat (SGW) on liver (**A**) and pancreas (**B**) tissues in the rats of different groups detected by HE staining. Scale bar: 10 μm.

Examination of HE-stained sections of the liver (Figure 3A) in the NDC group showed the typical architecture of the hepatic lobules. The hepatocytes form branches and anastomosis cords radiating from the central vein. In contrast, the DC group showed degenerative changes in the hepatocytes. The cells of the hepatic lobules possessed many vacuoles giving them a foamy appearance. The LGW group showed a hepatic degenerative change compared with the DC group, while in the HGW group the degenerative changes were reduced, presenting just few vacuoles, and becoming similar to the liver architecture of the NDC group.

The HE staining of the pancreas (Figure 3B) in the NDC group displayed a uniform arrangement of the pancreatic structure, with the pancreas islets containing several pancreatic β-cells. On the other hand, the pancreatic islet in the DC group showed remarkable damages compromising and deforming its shape with a reduction in the pancreatic β-cells content. The two SGW treatment groups partially recovered the damaged status and increased the number of pancreatic β-cells. 

## 4. Discussion

Based on this T2DM model of high-fat diet intake and treatment with STZ, our results showed for the first time that SGW consumption improved the glucose and lipid profile in the experimental T2DM rats by scavenging the free radicals.

Oxidative stress plays a crucial role in the pathogenesis of the T2DM and cardiovascular diseases. Persistent hyperglycemia causes an increase in the production of ROS through glucose auto-oxidation to induce oxidative stress [19]. Consumption of grain cereals is highly associated with reduced incidence of these diseases [20]. This may be due to a wide range of bioactive components with antioxidant effect, such as dietary fibers and phytochemical components in the grains. The novel variety wheat we used in our experiment, the SGW, did not present any significant difference in proteins, starch, fibers and fat content compared with the common wheat (data not shown). However, our results displayed a higher amount of total flavonoid and anthocyanin compared with the common wheat, as well as several biological functions such as the ability to decrease LDL-C, and remove ROS to prevent and/or efficiently treat oxidative stress-related diseases [7]. Indeed, our data showed a strong correlation between the SGW diet and the antioxidant activity through the improvement of the SOD and GSH-Px activities, which are considered as markers of the antioxidant system in the organism [11]. SGW diet indeed exerted its beneficial effects by scavenging the free radicals thanks to its higher content of cereal antioxidants. Moreover, the diet supplementation with SGW exhibited a clear hypoglycemic effect, which is in agreement with the effect of many cereal grains, such as barley and whole wheat [15,21]. These results demonstrated that the natural antioxidants in the grain could chelate metals as well as inhibit the free radicals by limiting the action of the lipoxygenase enzyme [22]. Additionally, SGW is functionally called “stay green” wheat since it actually maintains its photosynthetic competence for a longer time compared to the common wheat. Thus this peculiarity confers to this wheat a remarkably increased antioxidant power [13]. This phenotype delayed the senescence of the plants during the grain filling stage and showed a better redox state due to a higher activity of the antioxidant enzymes [12]. These differences may also affect the composition of the grain wheat. However, some epidemiological studies [23] showed no significant effects after the whole grain intake on insulin sensitivity and lipid mechanism, probably because of the short intervention time of only 6 weeks. Moreover, the different subjects and the food types are also important factors that may affect the results.

T2DM leads to glucose and fatty acids metabolic disorders. The typical symptoms of T2DM are represented by a higher need of food and water intake associated with the loss of body weight [24,25]. In the present study, the control rats constantly increased their body weight, whereas diabetes rats increased their body weight only after a high-fat diet intake. Subsequently, the body weights decreased due to the decrease in glucose metabolism and increase in fat metabolism [5]. SGW ameliorated these parameters at the end of the intervention period, indicating a regulatory effect on these metabolic disorders. Except the body weight, SGW was also able to regulate food and water intakes to reach the values showed by the NDC groups.

The loss of the β-cell function is a key event in the pathogenesis of diabetes. The insulin level and the insulin resistance both represent the main cause of inducing the hyperglycemia by destroying the structure of the pancreas and the β-cell function [26,27]. The β-cells are extremely sensitive to oxidative stress due to their low antioxidant abilities and their increased sensitivity to apoptosis [28]. The increased free radical production triggers β-cells injury through K_ATP_ pathway and up-regulation of the activity of the antioxidant enzymes, such as GSH-Px, SOD, and catalase. Our results showed that SGW was able to restore the normal status of the destroyed islets probably thanks to its high antioxidant content, especially flavonoid and anthocyanin. Therefore, SGW rich diet might be a promising strategy to prevent oxidative damages of the pancreatic islets for transplantation [29]. Furthermore, diabetes leads to a progressive accumulation of lipid metabolites. TG, TC, LDL-C, HDL-C and NEFA are considered as important biomarkers of hyperlipidemia [4]. Our results also confirmed that the SGW showed a strong hypolipidemic effect as well as a hypoglycemic effect through the reduction of TG, TC, LDL-C and NEFA except for HDL-C. However, the increasing amount of SGW feeding dose did not result in an enhanced effect, probably because the low dose of SGW was already enough to reduce the T2DM in the rat model. The abnormal blood lipid content could also represent the main factor in the pathogenesis of liver damage [30]. The SGW consumption could reverse most of the histological changes in the liver of the diabetes group. Based on some reports, the diabetic animals exhibited a reduction in the antioxidant ability, thus compromising the lipid metabolism function [31,32]. Since SGW contains many antioxidants, especially a high content of total flavonoid and total anthocyanin, it could mediate the metabolism of the lipids in diabetic rats via the support of the antioxidant ability and thus efficiently fight against hyperlipidemia. However, the specific molecular mechanism of SGW on diabetes needs further investigations.

## 5. Conclusions

The present results showed that SGW consumption reversed most of the pathological changes of the T2DM rats due to a high-fat diet feeding and STZ injection. The beneficial effects of SGW may be due to its strong antioxidant abilities thanks to its high content of total flavonoid and anthocyanin. Therefore, SGW possessed a significant anti-hyperglycemic effect and may represent a valid *in vivo* anti-oxidative dietary supplement for type 2 diabetes patients.
